# Breast Milk Conferred Immunity to Infants Against COVID-19

**DOI:** 10.7759/cureus.42075

**Published:** 2023-07-18

**Authors:** Riya Mary Richard, Giorgi Maziashvili, Mai Tran, Isabel Ramos, Anusha S Laxman, Nino Didbaridze

**Affiliations:** 1 Faculty of Medicine, Ivane Javakhishvili Tbilisi State University, Tbilisi, GEO; 2 Faculty of Medicine, Tbilisi State Medical University, Tbilisi, GEO; 3 Faculty of Medicine, Washington University of Health and Science, San Pedro, BLZ; 4 Facultad de Medicina y Cirugía, Universidad Católica de Honduras, Tegucigalpa, HND; 5 Department of Pediatrics, Bayshore Pediatrics, Houston, USA; 6 Department of Immunology, Tbilisi State Medical University, Tbilisi, GEO

**Keywords:** respiratory infections, breast milk, infant health, covid-19, breastfeeding

## Abstract

Coronavirus disease 2019 (COVID-19), caused by the severe acute respiratory syndrome coronavirus 2 (SARS-CoV-2), has notably impacted healthcare systems and everyday life worldwide. Regulatory authorities have approved the emergency use of SARS-CoV-2 vaccines due to the rapid spread of the virus. However, during vaccination testing, pregnant and breastfeeding women were initially excluded, leading to a lack of evidence-based recommendations. When taking the COVID-19 pandemic into account, breastfeeding has emerged as a potential defense mechanism against this infection due to its numerous benefits for newborns. Human breast milk contains immunoglobulins (IgA, IgG, and IgM), lactoferrin, and various cells that play an inevitable role in the newborn's protection against respiratory infections and immune system development. Various studies have highlighted that the onset and severity of respiratory infections in infants can be reduced through breastfeeding, and the effects are noticeable during the first six months of life and that breast milk also has the potential to enhance mucosal immunity and promote a diverse microbiome, reducing the risk of asthma, allergies, and enteric diseases through the provision of specific antibodies and immunological factors. Researchers have indicated that breastfeeding mothers who contracted and recovered from COVID-19 or received vaccination passed protective antibodies to their infants through breast milk. Although rare cases of detection of SARS-CoV-2 RNA in breast milk have been reported, the virus has not been cultured from these samples, suggesting a low risk of transmission to the breastfed baby. However, further research is essential to understand the extent of protection provided by breast milk against COVID-19 and the potential effect of distinct phases of lactation. Nonetheless, the current evidence supports the benefits and safety of breastfeeding during the pandemic. With appropriate safety measures, promoting breastfeeding can contribute to the overall health and well-being of infants during the phase of COVID-19.

## Introduction and background

The global outbreak of coronavirus disease 2019 (COVID-19), caused by the new coronavirus named severe acute respiratory syndrome coronavirus 2 (SARS-CoV-2), has had a tremendous impact, with millions of confirmed cases and high fatality rates, putting healthcare systems, economics, and daily life as we know it under strain [1]. Considering the rapid spread and burden of COVID-19, regulatory authorities approved SARS-CoV-2 vaccines for emergency use [2]. Several technological platforms exist, including traditional techniques such as complete live-attenuated and inactivated viral vaccines, protein subunit vaccines, and virus-like particles (VLPs). In addition, previously unexplored technologies for approved human vaccinations are being investigated. Examples of these include nucleic acid vaccines (mRNA and DNA), replicating and non-replicating viral and bacterial vectors, and modified antigen-presenting cells and T cells [3,4]. Unfortunately, pregnant and breastfeeding women were excluded from the early clinical studies of these vaccinations. As a result, the lack of data on this specific demographic has hampered the development of evidence-based recommendations, ultimately resulting in the exclusion of pregnant women from vaccine rollout plans [5,6]. Breastfeeding has been recognized as an important component of newborn health, with several advantages for both the baby and the mother. Following the COVID-19 pandemic, new data have revealed that breastfeeding may play a vital role in preventing infants from this unique viral illness. According to the Lancet Breastfeeding Series (2016), promoting mother-feed could avoid over 823,000 child deaths yearly. Breast milk ensures a 64% decrease in diarrhea morbidity, 74% in respiratory syncytial virus (RSV) pathogenicity, and 72% in hospitalization [7]. These findings highlight the protective effects of nursing in the context of the COVID-19 pandemic. Concerns have been raised about the transmission of COVID-19 from mother to child; however, current research has provided insight into the extraordinary protective effects of breastfeeding [8-10].

By analyzing current research findings, we aimed to explore how breast milk provided newborns with immunity against COVID-19, considering various methods, components, and potential consequences for infant health and illness prevention. Emphasizing the relevance of breastfeeding following a pandemic, we reviewed 55 articles published in the last 27 years using PubMed and ScienceDirect as search databases. Our analysis aimed to offer valuable insights to help healthcare professionals make informed recommendations about breastfeeding during this disease course.

## Review

Breast milk and the infant’s immune system

Human breast milk (HBM) is a multifaceted fluid that is essential for the transfer of nutrients and immunity, contributing to the short and long-term development and growth of newborns. Although the newborn's immune system is exposed to the mother's microbial flora during pregnancy, the infant's microbial environment undergoes abrupt changes during and after birth, making the infant highly vulnerable to illnesses. The components of breast milk assist newborns in developing immunocompetence and provide active and passive immunity [11]. With the rapid evolution of pathogens such as SARS-CoV-2 and the infant's immature immune system, infants rely on defense factors from their mothers. Immune transfer is achieved through the transplacental transport of immunoglobulin G (IgG) antibodies during the fetal period and the transport of immunoglobulin A (IgA) antibodies through breast milk after birth [12]. HBM contains nutritional substances, comprising approximately 88% water, and solid components comprising 7% (60-70 g/L) carbohydrates, 3.8% (35-40 g/L) fat, and 1% (8-10 g/L) protein, including macronutrients, vitamins, minerals, and also bioactive compounds [13]. The bioactive compounds in HBM, such as enzymes, hormones, specific proteins, polyamines, nucleotides, and oligosaccharides, have a biological effect on the gastrointestinal, vascular, nervous, and endocrine systems. They play a vital role in the growth of a newborn and are collectively referred to as "milk trophic factors" [14]. The production of breast milk occurs in three stages: colostrum, transitional milk, and mature milk [15]. Colostrum is the milk produced in the first five days following birth. It is rich in bioactive factors, including IgA, lactoferrin (LF), epidermal growth factor (EGF), transforming growth factor-beta (TGF-β), colony-stimulating growth factor, leukocytes, protein, human milk oligosaccharides, and antioxidants [16]. Transitional milk is produced from the fifth day up to two weeks, and maturation of the milk continues at least until four to six weeks postpartum, referred to as “mature milk.” Colostrum contains 90% IgA, 8% IgM, and 2% IgG, which is identical to mature breast milk. Additionally, the IgA and IgM present in the colostrum are in secretory form (sIgA and sIgM) connected to the J-chain and secretory component. The major cell types present in colostrum are leukocytes, such as macrophages expressing interferon alpha (IFNα), polymorphonuclear neutrophils, monocytes, T cells expressing CD45RO, cytotoxic T cells, dendritic cells (DCs), and a small proportion of B cells; these leukocytes account for about 10-70% of total cells [11]. Transitional milk and mature milk have a lower leukocyte count compared to colostrum, but they contain an increased number of IgAs and trophic factors like TGF-β. This difference is crucial for the continuous transfer of passive maternal immunity to newborns [17]. The evolution of colostrum to transition milk and then finally to mature milk during lactation presents gradual alteration to the content of milk, which is appropriate for the time-affected development of the infant [18]. The table below (Table 1) describes the main components of HBM with their respective functions in the immune system [19].

**Table 1:** Main components of human breast milk and their respective functions in the immune system IgA, immunoglobulin A; IgG, immunoglobulin G; IgM, immunoglobulin M; MFGE8, milk fat globule-EGF factor 8; MIF, macrophage migratory inhibitory factor; GCSF, granulocyte colony-stimulating factor; IL, interleukin; Th1, type 1 T helper; IFNγ, interferon-gamma; TGFβ, transforming growth factor beta; TNFα, tumor necrosis factor alpha; TNFR, tumor necrosis factor receptor; BMI, body mass index; VEGF, vascular endothelial growth factor; EGF, epidermal growth factor; HB-EGF, heparin-binding epidermal growth factor-like growth factor; IGF, insulin-like growth factor; RBC, red blood cell; NGF, nerve growth factor; MUC1, mucin 1; MUC4, mucin 4; HMOS, human milk oligosaccharides.

Immunoglobulins	
IgA/secretory immunoglobulin A (sIgA)	Pathogen binding inhibition
IgG	Anti-microbial; activation of phagocytosis (IgG1, IgG2, IgG3); anti-inflammatory response to allergens (IgG4)
IgM	Agglutination; complement activation
Anti-microbial	
Lactoferrin	Acute phase protein; chelates iron; anti-bacterial; anti-oxidant
Lactadherin/MFGE8	Anti-viral; prevents inflammation by enhancing phagocytosis of apoptotic cells
Cells	
Stem cells (mammary stem cell, hematopoietic stem cell, mesenchymal stem cells, and pluripotent stem cell)	Regeneration and repair
Chemokines	
MIF	Prevents macrophage movement and increases the anti-pathogen activity of macrophages
GCSF	Trophic factors in intestines
Cytokines	
IL-6	Stimulates the acute phase response; B-cell activation; pro-inflammatory
IL-7	Improves thymic function and increases its size
IL-8	Recruitment of neutrophils; pro-inflammatory
IL-10	Repressing Th1-type inflammation; induction of antibody production; facilitation of tolerance
IFNγ	Pro-inflammatory; stimulates Th1 response
TGFβ	Anti-inflammatory; stimulation of T cell phenotype switch
TNFα	Stimulates inflammatory immune activation
Cytokine Inhibitors	
TNFR I and II	Inhibition of TNFα; anti-inflammatory
Hormones	
Somatostatin	Regulation of gastric epithelial growth
Calcitonin	Development of enteric neurons
Metabolic hormones	
Adiponectin	Reduction of infant BMI and weight; anti-inflammatory
Leptin and ghrelin	Regulation of energy conversion and infant BMI; appetite regulation
Growth factors	
VEGF	Promotes angiogenesis and tissue repair
EGF	Stimulates cell proliferation and maturation
HB-EGF	Protective against damage from hypoxia and ischemia
IGF	Stimulation of growth and development; increases RBCs and hemoglobin
NGF	Promotion of neuron growth and maturation
Erythropoietin	Erythropoiesis; intestinal development
Mucins	
MUC1 and MUC4	Prevents infection by viruses and bacteria
Oligosaccharides and glycans	
Glycosaminoglycans	Anti-infectious
Gangliosides (GM1, GM3, and GD3)	Brain development; anti-infectious
HMOS	Probiotic; stimulating beneficial colonization; reducing colonization with pathogens; reducing inflammation

Recent studies have provided evidence of the presence of anti-SARS-CoV-2 IgA antibodies in the colostrum of pregnant patients with COVID-19 infection [20,21]. This presence of anti-SARS-CoV-2 IgA has been associated with a reduced frequency of clinical symptoms in newborns [21]. Considering the significant increase in immunoglobulin expression in response to SARS-CoV-2 infection, it is worth exploring this topic further. The origin of IgA in human milk is from the gastrointestinal system of the mother, a process known as the “entero-mammary gland pathway” [22]. When a pathogen enters the mother's upper airway, the lymphoid cells in the Peyer's patches (PPs) capture the pathogen. Antibody production for specific antigens relies on the sampling by Peyer's patch M cells, which are then processed by antigen-presenting cells such as DCs. Ultimately, DCs present the antigens to T lymphocytes, resulting in T cell activation and B cell class switching in gut-associated lymphoid tissue (GALT), mesenteric lymph nodes, and lamina propria [23]. Circulating B cells then migrate to various sites in the body where local slgA production occurs, such as the mammary glands, cisterna chyli, thoracic duct, vascular circulation, mucosal membranes, and exocrine glands [17]. Within the mammary epithelial cell, the B cells migrate to the basolateral surface or serosal side and produce dimeric IgA [20,24]. The dimeric IgA is bound by a polymeric immunoglobulin receptor (pIgR), undergoes endocytosis, and acquires a carbohydrate chain in a process called glycosylation. At the apical side of the mammary epithelial cell, the pIgR undergoes proteolytic cleavage, while the secretory component of pIgR remains bound to the IgA antibody and sIgA is released [20,25]. sIgA is resistant to digestion during lactation when a newborn consumes milk [26]. This is achieved by the secretory component of IgA and is essential for the milk antibodies to survive the gastric environment and be transferred to the infant effectively [27]. Finally, sIgA accumulates in the intestine of the newborn, binding to antigens on the pathogen and providing immunity [26]. sIgA synthesis is aided by several cytokines such as IL-4, TGF-β, IL-5, IL-6, and IL-10. TGF-β and IL-10 are also vital in maintaining mucosal tolerance. The breast milk sIgA that is transferred to the newborn provides mucosal defense mainly by intracellular neutralization, viral excretion, and immunological exclusion (protecting the intestinal epithelium from microorganisms and their toxins) [20]. Furthermore, various studies have demonstrated that numerous factors like gestational age, body mass index, diet, lifestyle and habits (maternal nutrition), maternal use of antibiotics, vaccination, geographic location, maternal pathologies, delivery route (vaginal and cesarean section), smoking, lactation phase, and maternal psychological stressors influence immunoglobulin production from breast milk [28-30]. Overall, it is important to note that breast milk serves as a powerful ally in bolstering the infant's immune system, providing an array of protective factors that contribute to the overall health and well-being of babies.

Breast milk and immunity against respiratory infections in infants

Breast milk plays a vital role in the immunological development of a child's early life, as it provides numerous immunological factors and cytokines that support immunity. Several studies have demonstrated that children who are exclusively breastfed have a lower incidence and severity of respiratory infections compared to those who receive additional forms of nutrition [31,32]. This reduced risk of infection is largely attributed to the increased diversity in the upper respiratory tract (URT) and gut microbiome, which is influenced by breastfeeding [33,34].

The colonization of the infant gut microbiome begins during birth when the newborn is exposed to the vaginal canal, the external environment, and the mother's external microbiome while nursing [33]. Maternal antibodies and immunoglobulins are retained by most children up to around six months of age, during which transplacental IgGs decrease, and the infant's immune system starts developing with the assistance of primarily IgA from breast milk [33,35]. Breastfeeding has also been associated with the production of interferons (IFN-α and IFN-γ) and IL-17, which contribute to its protective effects against the development of lower respiratory tract infections (LRTIs), asthma, and allergic rhinitis in infancy and early childhood [34,36].

Breast milk is tailored to meet the needs of children throughout each stage of life due to the various cytokines and immunological factors it contains [31]. For instance, placental IgG is a primary contributor to the lowered risk of respiratory infection in children aged three to six months, since it remains in the immune system up to six months of age. After this point, IgA from breast milk begins to predominate within mucosal sites and becomes a main source of immune support [33]. This is believed to be why children who are primarily or exclusively breastfed show decreased incidence of mucosa-related illnesses such as respiratory infections, allergies, and enteric diseases [32,33]. Since newborns have limited gut biodiversity, they are highly susceptible to different microbial species and various forms of mucosal infections. Early choices such as feeding practices, antibiotic use, and mode of delivery can significantly impact the development of the infant's immune system. For example, children delivered via C-section have been historically more likely to develop asthma, allergies, and other mucosal complications later in life [33]. Additionally, breastfeeding has been associated with a lower risk of chronic non-communicable diseases such as obesity, asthma, cancer, and diabetes later in life [31]. As the immune system matures and stabilizes its colonization around two to three years of age, appropriate environmental exposure becomes crucial for the child's development [33].

RSV is a leading cause of LRTIs and hospitalization among young infants in middle-to-low-income families. Over 6,000 studies have found that non-breastfed children or those with limited access to breast milk early in life are more likely to contract RSV and experience more severe infections. Breastfed infants, regardless of additional supplementation, tend to have shorter hospital stays and require less respiratory intervention when infected with RSV and other LRTIs [32]. This protective effect is attributed to the mucosa-specific antibodies present in breast milk, in contrast to the systemic immunoglobulins (particularly IgG) that are passed transplacentally to the fetus during pregnancy. It is worth noting that IgG plays a significant role in protecting preterm infants, who are most vulnerable to severe RSV disease, from experiencing a more severe course of illness than non-breastfed infants. Since many childhood respiratory infections lack vaccines, various organizations, including the Centers for Disease Control and Prevention, United Nations Children's Fund, and World Health Organization, recommend breastfeeding within the first hour of life and exclusive breastfeeding for the first six months, followed by supplementation with liquid or solid foods up to two years, to mitigate the risk and duration of infections in young children [36-39].

Interestingly, some studies have reported mixed or negative correlations between breast milk and the nasal biome. While breastfed children have shown a reduced incidence of diseases such as gastroenteritis, conjunctivitis, laryngitis, and tracheitis, in some instances, results indicated an increase in the occurrence of the common cold [31]. Since research into the nasal microbiome is still limited, the exact cause for this discrepancy is unknown. Researchers speculate that oral feeding alters the gut microbiome more immediately and extensively, while the early nasal biome is predominantly shaped by the infant’s environmental exposures [40]. However, despite these few studies with contradictory results, recent research has provided positive and correlational findings regarding the transfer of COVID-19 immunity from mother to child through breast milk [35]. These studies have demonstrated that maternal antibodies against SARS-CoV-2 can be transferred to infants at detectable levels when the mother contracts and recovers from the virus during pregnancy and subsequently receives the vaccine. In many cases, the levels of serum antibodies positively correlate with those passed through breast milk, with a predominant presence of IgA, indicating its specific role in providing infant immunity against the virus. Moreover, one study revealed that these antibodies could persist in breast milk for up to 300 days [35,40]. However, due to the emergence of different SARS-CoV-2 variants, further research is ongoing to determine whether the conferred immunity from breast milk can elicit an adequate response against each subtype in infants [35].

Breastfeeding and COVID-19

Breast milk, renowned for its exceptional qualities in providing infant nutrition, contains a wide range of antimicrobial components, including virus-neutralizing antibodies [41,42]. These antibodies offer numerous health benefits to newborns, protecting them from enteric and other diseases, thus reducing diarrhea cases among infected children [42]. The presence of abundant immunoglobulins in colostrum and milk plays a crucial role in the transfer of passive immunity from mother to child, establishing a vital immunological connection. These secretions not only contain immunoglobulins but also other immune factors that can benefit various species, including humans [42]. While breastfeeding is generally beneficial, it is important to acknowledge the potential risks associated with the transmission of viruses like human T lymphoma virus (HTLV) or HIV to newborns [43].

A comprehensive meta-analysis by Boostani et al. explored the risks associated with specific durations of breastfeeding among infected mothers and infants [43]. Their study revealed that short-term lactation (less than six months) is not linked to an increased risk of HTLV I virus transmission from mother to child through breast milk. However, mothers who continue breastfeeding beyond this duration do significantly increase the risk compared to those who discontinue breastfeeding earlier or avoid nursing altogether [43]. On the other hand, the American Academy of Pediatrics has recommended that infants who have received approved hepatitis B immune globulin (HBIG) and the hepatitis B vaccine should not consider hepatitis B virus infection a contraindication to breastfeeding [44]. It is crucial to recognize that breast milk is not homogeneous, and its composition changes during lactation. Further research is needed to determine whether the risk of virus transmission varies with the different stages of lactation.

Moreover, breast milk, with its diverse composition varying between pregnancies and mothers, continues to be recognized as the optimal source of nutrition for infants, offering numerous well-documented immunological benefits [45]. Nonetheless, to fully comprehend the precise role of immune cells, such as effector memory cells, and innate immune cells like macrophages, in safeguarding newborns against infections and immunological-related disorders, further research is necessary [45].

A study investigating the presence of SARS-CoV-2 in breast milk found viral RNA (vRNA) through a reverse transcription-polymerase chain reaction in milk samples obtained six to 10 days after the mother's initial positive COVID-19 test result [46]. The research involved the examination of 64 human milk samples collected from 18 women who had recently contracted COVID-19, and it detected vRNA in the milk sample of one symptomatic woman [46]. However, there is currently no evidence to suggest that an infectious virus is present in breast milk or that breastfeeding is a risk factor for transmitting the infection to newborns [46]. While no active virus has been detected in breast milk, studies have shown that Holder pasteurization effectively deactivates SARS-CoV-2 in breast milk [47]. There have been rare cases of detecting SARS-CoV-2 RNA in breast milk samples of women with confirmed infection, but even when detected, longitudinal follow-up studies have shown that it is unlikely to infect the breastfed baby. The presence of virulent RNA was temporary and no cultured SARS-CoV-2 was found in any of the specimens [48]. In a retrospective review by Chambers et al., limited data were found regarding the potential transmission of COVID-19 from mother to infant through breast milk [49]. Although SARS-CoV-2 RNA was found in one milk sample from an infected woman, the viral culture for that sample was negative [49]. This suggests that the detected RNA does not indicate the presence of a replication-competent virus, meaning that breast milk cannot be a source of infection for infants [49].

According to a study on the effect of maternal vaccination on infants, SARS-CoV-2 enters cells through the angiotensin-converting enzyme 2 (ACE2) receptor, which is prevalent in the epithelium of the human lungs, colon, and small bowel [50]. Specific IgG and IgA antibodies against SARS-CoV-2 found in the breast milk of vaccinated individuals could potentially interfere with the interaction between viral ACE2 receptors and the infant's intestinal mucosa, providing protection [50]. Additionally, even before immunization, HBM has been found to neutralize the pseudovirus in vitro due to the cross-reactivity of antibodies to different coronaviruses [51]. Breast milk contains a wide range of immune system-defending molecules, and an increase in milk neutralization potential was observed in 50% of samples collected six months after vaccination, despite a decrease in antibody levels. This may be attributed to the specialization and maturation of antibodies in milk, similar to what has been observed for COVID-19 IgG in blood plasma [52].

Breastfeeding has known benefits in protecting newborns, infants, and children from morbidity and mortality [53]. Research also suggests that breastfeeding may enhance vaccine responses and actively stimulate the infant's immune system by transferring anti-antibodies, T and B lymphocytes, cytokines, and growth factors through breast milk [54]. The direct transmission of anti-infective agents and antibodies, along with the ongoing immunological memory transfer contribute to the effectiveness of breastfeeding in protecting against infections [54]. These findings provide compelling evidence that breastfeeding from mothers infected with SARS-CoV-2 poses no risk to infants. The benefits of breastfeeding outweigh the potential risks of COVID-19, and it may even have a protective effect for both the newborn and the mother.

## Conclusions

Following the COVID-19 outbreak, numerous research studies have been conducted, shedding light on the potential role of breastfeeding in preventing infants from the illness. The abundance of immunoprotective characteristics found in breast milk, coupled with the lower incidence and severity of infections in breastfed children, suggests that breastfeeding may play an important role in protecting infants from COVID-19. The presence of antibodies, particularly IgA, in breast milk, can provide passive and active immunity to the infant, thereby aiding in the prevention of respiratory diseases. Recent findings indicate that breast milk from mothers who have been vaccinated or recovered from a SARS-CoV-2 infection contains maternal antibodies against the virus, offering acquired protection for the newborn and a low risk of infection. Although rare instances of detecting SARS-CoV-2 RNA in breast milk samples have been reported, the virus has not been successfully cultured from these samples, suggesting a minimal risk of transmission to breastfed babies. Nevertheless, additional research is required to comprehensively understand the extent of protection provided by breast milk against COVID-19 and the potential impact of different stages of lactation. Large-scale cohorts are needed to investigate the timing of viral shedding in milk and the neutralizing capacity of transmitted antibodies to draw appropriate conclusions on breastfeeding-acquired immunity against COVID-19. However, based on the current evidence, breastfeeding is considered safe and beneficial for both newborns and mothers during the ongoing pandemic. Promoting breastfeeding, along with appropriate safety measures, can contribute to the overall health and well-being of infants in the face of COVID-19.
